# Preparation, Characterization and Dermal Delivery of Methadone

**DOI:** 10.3390/pharmaceutics11100509

**Published:** 2019-10-02

**Authors:** Chin-Ping Kung, Bruno C. Sil, Jonathan Hadgraft, Majella E. Lane, Bhumik Patel, Renée McCulloch

**Affiliations:** 1 Department of Pharmaceutics, UCL School of Pharmacy, 29-39 Brunswick Square, London WC1N 1AX, UK; jonathan.hadgraft@btinternet.com (J.H.); majella.lane@btinternet.com (M.E.L.); 2 School of Human Sciences, London Metropolitan University, 166-220 Holloway Road, London N7 8DB, UK; b.dasilvasildossantos@londonmet.ac.uk; 3 Great Ormond Street Hospital for Children NHS Foundation Trust, Great Ormond Street, London, WC1N 3JH, UK; bhumik.patel@gosh.nhs.uk (B.P.); renee.mcculloch@gosh.nhs.uk (R.M.)

**Keywords:** dermal delivery, porcine skin, in vitro permeation, methadone, pain

## Abstract

The use of methadone for the management of pain has received great interest in recent years. Currently, oral and intravenous formulations are available for clinical use. Dermal delivery represents an attractive alternative route of administration for this drug as it is associated with comparatively fewer side effects. The first stage of the work was the preparation of methadone free base as this form of the drug is expected to permeate the skin to a greater extent than the hydrochloride salt. Subsequently the molecule was characterized with Nuclear Magnetic Resonance (NMR) and thermal analysis, the distribution coefficient was determined and solubility studies were conducted in a range of solvents. In vitro permeation and mass balance studies were conducted under finite dose conditions (5 μL/cm^2^) in porcine skin. The results confirmed the more favorable penetration of methadone free base compared with the salt. The highest cumulative amount of methadone (41 ± 5 μg/cm^2^) permeated from d-limonene (LIM). Ethyl oleate (EO), Transcutol^®^ P (TC) and octyl salicylate (OSAL) also appear to be promising candidate components of dermal formulations for methadone base. Future work will focus on further formulation optimization with the objective of progressing to evaluation of prototype dosage forms in clinical trials.

## 1. Introduction

Neuropathic pain can be caused by injuries, surgery, chemotherapy and a number of disease conditions such as diabetes mellitus, cancer and human immunodeficiency virus infection [1]. It can also arise as a direct consequence of a lesion or disease affecting the peripheral nervous system or the central nervous system [2]. Patients’ overall health-related quality of life is greatly impacted by neuropathic pain [3] and it is estimated to affect 7–10% of the general population in Europe [4]. The available current knowledge in terms of causes, diagnosis and treatment for peripheral neuropathic pain is more advanced than that for pain associated with the central nervous system [5]. However, to date, there are still very few effective treatments available for management of neuropathic pain. 

Methadone has traditionally been used in the management of opioid dependence. However, there is increasing interest in the repurposing of methadone as a cost-efficient treatment for neuropathic pain [6,7]. Morley, et al. [8] reported the efficacy of oral methadone for the management of neuropathic pain in a double-blind randomized controlled crossover trial. 19 patients were enrolled in the trial and a statistically significant (*p* < 0.05) improvement in pain relief was reported. Methadone is not only a potent μ-opioid receptor agonist but also a non-competitive *N*-Methyl-d-aspartate (NMDA) receptor antagonist [9]. The combination of NMDA receptor antagonism and opioid receptor agonism induced by methadone is thought to result in enhanced analgesic efficacy. Animal studies have demonstrated that the antinociception effect of methadone is mediated by both opioid agonism and NMDA receptor antagonism in neuropathic pain [10]. The peripheral effect of morphine, a μ-opioid receptor agonist, was enhanced by combined administration of (+)-HA966, an NMDA receptor antagonist, in a rat model of neuropathic pain [11]. In addition, Stoetzer, et al. [12] also suggested that methadone is an unselective blocker of a number of voltage-gated sodium (Nav) channels including Nav1.2, Nav1.3, Nav1.7, and Nav1.8 with comparable potency to bupivacaine. These studies suggest that methadone is a promising candidate to be developed for the management of peripheral neuropathic pain.

Dermal delivery of drugs offers a number of advantages compared with other routes of administration. Constant drug levels in the plasma can be achieved with a decrease in dosing frequency. Moreover, the avoidance of first pass metabolism results in less intra- and inter-patient variability. Dermal delivery should also provide higher concentrations of methadone in skin tissues, thus targeting pain at its source. The peripheral actions of methadone also suggest a lower concentration of methadone can be delivered via the skin for pain relief compared with oral administration. To our knowledge, there are only three published papers that have examined the in vitro skin absorption of methadone. Fullerton, et al. [13] examined the permeation of methadone in human cadaver skin from simple ethanol solutions. Ghosh and Bagherian [14] also investigated the delivery of methadone free base from a transdermal patch in human skin. Recently, Muñoz, et al. [15] reported in vitro permeation studies using porcine skin to evaluate the efficiency of methadone hydrochloride patches. No studies have examined the in vivo delivery of this compound through the skin. 

The aims of the present work were (i) to prepare and characterize methadone free base and (ii) to identify effective solvents for the dermal delivery of methadone. A concentration of 5% *w/v* methadone base solutions was selected based on available formulations for oral use. A range of solvents, spanning different physicochemical parameters such as polarity and solubilization properties, were selected as candidate vehicles. These solvents are generally recognized as safe (GRAS) and are already used in other topical and transdermal dosage forms. 

## 2. Materials and Methods

### 2.1. Materials

Methadone hydrochloride, tripropylene glycol (TriPG), dipropylene glycol (DiPG), 1-octanol, 1,3-butanediol (1,3-BD), Brij™ O20, isopropyl myristate (IPM), octyl salicylate (OSAL), oleic acid (OA) and ethyl oleate (EO) were purchased from Sigma-Aldrich, Dorset, UK. Propylene glycol (PG), ethyl acetate, d-limonene (LIM), high performance liquid chromatography (HPLC) grade water, acetonitrile, orthophosphoric acid, 85+%, sodium dihydrogen phosphate, sodium hydrogen phosphate and sodium hydroxide were supplied by Fisher Scientific, Loughborough, UK. Chloroform-*d* (CDCl_3_) was purchased from Cambridge Isotope Laboratories Tewksbury, MA, USA. Labrafac™ lipophile WL 1349 (medium-chain triglycerides of caprylic and capric acids, WL 1349), Transcutol^®^ (TC), propylene glycol monocaprylate (PGMC) Type II and propylene glycol monolaurate (PGML) Type II were gifts from Gattefossé, St. Priest, France. Phosphate buffered saline (PBS) (pH 7.3 ± 0.2 at 25 °C) was prepared using Dulbecco A tablets supplied by Oxoid, Cheshire, UK. Full thickness porcine ear skin was obtained from a local abattoir.

### 2.2. Preparation of Methadone Base

Methadone hydrochloride (1.730 g, 0.005 mol) was placed in a dry 250 mL round-bottom flask with a Teflon coated magnetic stirrer. 20 mL of HPLC grade water was added and the mixture was stirred for 10 min. The pH was then adjusted to 11.0 with 1 M sodium hydroxide solution. The newly formed precipitate was stirred for 30 min. 100 mL of ethyl acetate was added to the suspension with stirring for 60 min at 25 °C. The organic layer was then collected, and the aqueous layer washed with 45 mL (3 × 15 mL) of ethyl acetate. All organic layers were collected together and dried with magnesium sulfate. Ethyl acetate was removed using a rotary evaporator (Heidolph, Schwabach, Germany) using a high vacuum line for 5 h. Structural characterization was conducted using proton nuclear magnetic resonance (^1^H-NMR) spectroscopy. The spectrum was obtained in chloroform-*d* using a Bruker Avance 500 MHz NMR spectrometer (Bruker Corporation, Billerica, MA, USA) and processed using MestReNova 11.0.4 (Mestrelab Research, Santiago de Compostela, Spain). The Fourier transform infrared (FTIR) spectra were obtained with a Bruker Alpha Spectrometer with a Platinum ATR accessory (Bruker Optics, Coventry, UK) in transmittance mode. Spectral analysis and instrument control were performed with OPUS software v.7.0 (Bruker Optics). The spectrum of each sample was measured by taking the average of 64 scans at a resolution of 2 cm^−1^ at room temperature. The reference background spectrum was recorded before collecting IR spectra.

### 2.3. Thermal Analysis

The melting points of methadone hydrochloride and methadone free base were measured using thermogravimetric analysis (TGA) and differential scanning calorimetry (DSC). Discovery TGA (TA instruments, Waters, LLC, USA) was used for the degradation temperature identification. Approximately 3 mg of sample was placed in a tared aluminium pan. A heating ramp of 10 °C/min to 550 °C was used during the analysis. A nitrogen flow of 25 mL/min was supplied to create an inert atmosphere around the sample. A DSC Q2000 (TA instruments) system was used to measure the melting point. The sample was weighed in a hermetic aluminium pan which was subsequently sealed with a hermetic aluminium lid using a Tzero press. An empty hermetic aluminium pan sealed with a hermetic aluminium lid was used as a reference. Sample and reference pans were heated with a heating ramp of 10 °C/min with nitrogen as the purge gas (50 mL/min).

### 2.4. HPLC Analysis

The HPLC system used in this study consisted of an Agilent G1322A degasser, G1311A quaternary pump, G1313A auto sampler, G1316A thermostat column compartment and G1315B diode array detector (Agilent Technologies, Palo Alto, CA, USA.). The software used to acquire and analyze the data was ChemStation^®^ for LC 3D, Rev. A. 09.03 (Agilent Technologies). Analysis was performed with a Luna^®^ Omega 5 µm PS C_18_ 150 × 4.60 mm column (Phenomenex, Macclesfield, UK), equipped with a universal HPLC guard column packed with a SecurityGuard™ C_18_ cartridge. The mobile phase consisted of 20 mM sodium phosphate buffer at pH 3 ± 0.2 and acetonitrile (60:40, *v/v*) and the flow rate was 1 mL/min. The column temperature and injection volume were set to 30 °C and 10 μL, respectively. Ultraviolet (UV) detection at 213 nm was employed. The HPLC method was validated in terms of specificity, linearity, accuracy, precision, limit of detection (LOD) and limit of quantification (LOQ) according to the International Conference of Harmonization guidelines [16]. Calibration curves in the concentration range of 0.5–100 μg/mL were constructed (r^2^ ≥ 0.99) and the LOD and LOQ values were 0.23 and 0.69 µg/mL, respectively.

### 2.5. Log D

The method used for Log D measurement was the shake flask method, adapted from the Organization for Economic Co-operation and Development (OECD) guidelines [17]. 0.2 M sodium phosphate buffer solutions were prepared at pH 6.0, 6.5, 7.0, 7.4 and 11.0 and mutually saturated with 1-octanol by slow-stirring (at 130 rpm) for 48 h at 25 °C. The solutions were equilibrated in a separation funnel for 24 h before separation. Methadone solutions in octanol were prepared at 0.1 and 0.5 mmol/L. These solutions were then mixed with the aqueous phase (sodium phosphate buffers) at three different ratios (1:1, 2:1 and 1:2). To reach equilibrium, the mixtures were rotated approximately one hundred times through 180° at room temperature. Before sampling, the mixtures were centrifuged at 25 °C and at 13,000 rpm for 30 min. Samples were taken from each phase with suitable dilution using methanol and analyzed using the HPLC method described in Section 2.4. 

### 2.6. Solubility and Stability

Stability studies of methadone free base in the Franz cell receptor medium (6% *w/v* Brij™ O20 in pH 7.4 PBS) and a range of neat solvents were conducted for 96 h. A known quantity of methadone free base was dissolved in the receptor medium or neat solvent. These solutions were placed in Eppendorf^®^ tubes and sealed with Parafilm^®^ before being placed in a shaker (VWR, Leicestershire, UK) at 32 ± 1 °C. Samples were taken at 0, 24, 48, 72 and 96 h. The samples were diluted when necessary and analyzed using the HPLC method described in Section 2.4.

The solubility parameter values (δ) of the solvents were calculated by the van Krevelen-Hoftyzer approach [18] using Molecular Modelling Pro^®^ Version 6.3.3 software (ChemSW, Fairfield, CA, USA). In total, 13 different single solvents were selected for solubility studies in order to ensure that solvents with a wide range of solubility parameter values were investigated. Solubility studies were also conducted with PBS and PBS with 6% Brij™ O20 (*w/v*) solution to confirm that sink conditions would be maintained during the in vitro permeation studies. 

For solubility determinations, an excess amount of methadone hydrochloride or free base was added to 0.5 mL of each solvent in Eppendorf^®^ tubes (*n* = 3). The tubes were sealed with Parafilm^®^ and placed on a rotator at 32 ± 2 °C for at least 48 h. These samples were then centrifuged at 12,000 rpm for 15 min at 32 ± 1 °C. The supernatant was suitably diluted to lie within the range of the calibration curve of the validated HPLC method, described in Section 2.4.

### 2.7. In Vitro Permeation and Mass Balance Studies

In vitro permeation studies were carried out as reported previously by Santos, et al. [19] using vertical glass Franz diffusion cells. Full thickness porcine ear skin was separated from the underlying tissue at room temperature and then stored at −20 °C before use. Skin integrity was assessed by measuring impedance at a frequency of 50 Hz before applying formulations [20]. The diffusion area was ~1 cm^2^ and was accurately measured before application of finite doses (5 μL/cm^2^) of 5% *w/v* methadone base solutions. The complete dissolution of methadone in all solvents was confirmed in the preparation of formulations. 6% *w/v* Brij™ O20 was added to the receptor phase, PBS pH 7.3 ± 0.2 to ensure the concentration of methadone never exceeded 10% of its solubility without affecting the integrity of the skin [21,22]. Sink conditions in this medium were therefore confirmed during experiments. Permeation studies were performed for 24 h at 32 ± 1 °C. 200 μL samples were withdrawn from the receptor compartment and replaced with 200 μL of fresh receptor medium at 0, 2, 4, 6, 8, 10, 12, 24 h.

Validated mass balance studies were performed after each permeation study. The skin surfaces were washed three times with 1 mL of methanol. The skin was then placed in Eppendorf^®^ tubes with 1 mL of methanol. The samples for skin extraction were placed in a shaker overnight to extract methadone. All Eppendorf ^®^ tubes were centrifuged at 13,000 rpm at 32 °C for 15 min before sampling. The supernatants were sampled and diluted where necessary and analyzed by HPLC.

### 2.8. Statistical Analysis

Microsoft Excel^®^ 2016 was used for data processing and the calculation of mean and standard deviation (SD). IBM^®^ SPSS Statistics^®^ 24.0 (IBM SPSS Statistics, Feltham, UK) was used for statistical analysis. The Shapiro-Wilk test was used to assess the normality of the data. If the *p* value calculated from the test was higher than 0.05, the data were assumed to be normally distributed. For normally distributed data (parametric data), the independent-samples t test and one-way analysis of variance (ANOVA) with Tukey’s HSD post hoc test were used to analyze two groups and ≥3 groups respectively. For non-normally distributed data (non-parametric data), the Mann-Whitney U test was used to test statistical significance between two groups and the Kruskal-Wallis one-way ANOVA test was performed to investigate statistical differences among different groups (≥3 groups). A probability of *p* < 0.05 was considered as a statistically significant difference.

## 3. Results and Discussion 

### 3.1. Characterization of Methadone Free Base with NMR and IR Spectroscopy

**Figure 1:**
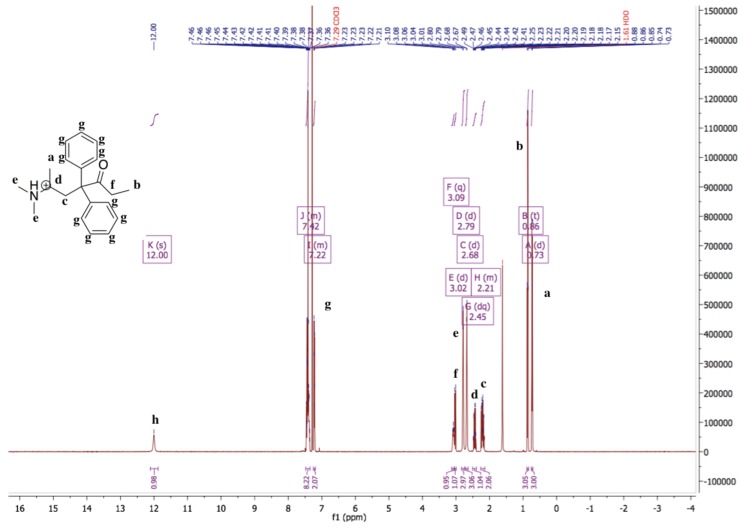
^1^H-NMR spectrum of methadone hydrochloride in chloroform-*d*. ^1^H-NMR (500 MHz, Chloroform-*d*) δ 12.00 (s, 1H), 7.48–7.35 (m, 8H), 7.25–7.19 (m, 2H), 3.09 (q, 1H), 3.02 (d, 1H), 2.79 (d, 3H), 2.68 (d, 3H), 2.45 (dq, 1H), 2.26–2.14 (m, 2H), 0.86 (t, 3H), 0.73 (d, 3H). A singlet at 12.00 ppm suggests the protonation of the tertiary amine.

**Figure 2:**
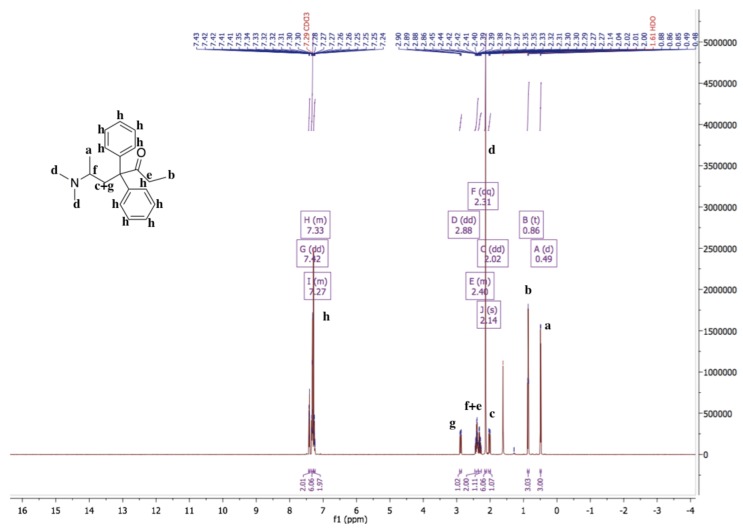
^1^H-NMR spectrum of methadone free base in chloroform-d. ^1^H-NMR (500 MHz, Chloroform-*d*) δ 7.42 (dd, 1.6 Hz, 2H), 7.36–7.30 (m, 6H), 7.28–7.24 (m, 2H), 2.88 (dd, *J* = 13.9, 5.5 Hz, 1H), 2.46–2.36 (m, 2H), 2.31 (dq, 7.2 Hz, 1H), 2.14 (s, 6H), 2.02 (dd, *J* = 13.9, 5.6 Hz, 1H), 0.86 (t, 3H), 0.49 (d, 3H). The ^1^H-NMR spectrum of methadone free base shows that there is no singlet around 12.00 ppm for the hydrochloride proton.

The ^1^H-NMR spectrum of methadone hydrochloride is shown in Figure 1. A singlet at 12.00 ppm suggests the protonation of the tertiary amine while the ^1^H-NMR spectrum of methadone free base shows that there is no singlet around 12.00 ppm for the hydrochloride proton (Figure 2). In addition, all spectral peaks were assigned for both methadone hydrochloride and free base. A water singlet at 1.61 ppm in both spectra may reflect small amounts of water present in the samples [23]. These results confirm the successful conversion of methadone hydrochloride to the free base. Methadone free base was obtained as a white powder, with a yield of 92%. The IR spectra of methadone free base and methadone hydrochloride also support the conclusion that methadone hydrochloride was successfully converted to methadone free base (Figure S1). Characteristic peaks for methadone were obtained for both methadone hydrochloride and free base; a broad band at 2399 cm^−1^ was assigned to the NH^+^ stretching of methadone hydrochloride.

### 3.2. Thermal Analysis

The thermogravimetric analysis of methadone hydrochloride and methadone free base is shown in Figure S3. The decomposition of methadone hydrochloride is evident from 167 to 261 °C (onset: 230.12 °C) while that of methadone free base occurs from 134 to 230 °C (onset: 194.35 °C).

**Figure 3:**
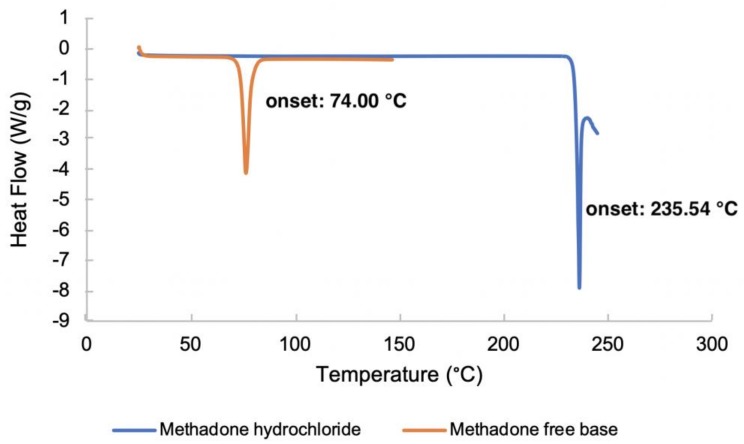
DSC thermograms of methadone hydrochloride and methadone free base.

The DSC thermograms of methadone hydrochloride and the free base are shown in Figure 3. The melting points of methadone free base and methadone hydrochloride were obtained as the onset temperatures. The melting point of methadone hydrochloride (235.54 °C) is very close to the degradation temperature of the compound. Only one endothermic event which occurs between 73.5 °C and 79.8 °C was observed for methadone free base. The obtained melting point of methadone free base is 74.00 °C which is consistent with the literature (76 °C) [24].

### 3.3. Log D Measurement

The predicted values of the Log P for methadone base from ChemAxon and ACD/Labs were 5.0 and 4.2, respectively. These values are higher than the experimental Log P value of 3.93 reported previously [25]. The measurement of the partition coefficient of an ionizable species should be made in the non-ionized form according to the OECD guidelines [17]. An appropriate buffer with a pH of at least one unit above the dissociation constant (pK_a_) of methadone (pK_a_ = 8.94) [26] should be used to ensure the compound is present as the free base. Hence, 20 mM pH 11 PBS was used in this study to measure the partition coefficient of methadone base. The value obtained in this measurement was 4.85 ± 0.03. The Log D values obtained at different pH values are listed in Table 1. The log D value at pH 7.4 is lower than the experimental value previously reported (Log D_7.4_ = 2.1) [27], however no details of the buffer or ionic strength used were given for this earlier measurement. 

### 3.4. Stability and Solubility

After 96 h stability studies at 32 ± 1 °C, the recovery values of methadone base in all solvents was >97%. The chemical stability of methadone free base over 96 h in the receptor medium for permeation studies and common solvents, including LIM, EO, IPM, WL 1349, OA, PGML, PGMC, TC, OSAL, TriPG, DiPG, PG, 1,3-BD and ethyl acetate, was confirmed (Figure S4).

**Figure 4:**
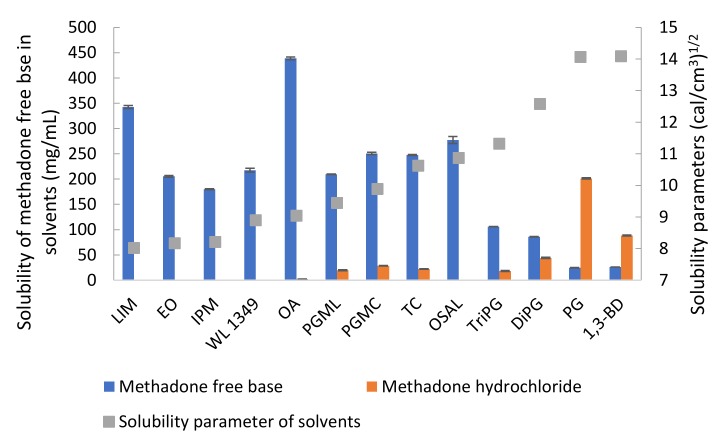
Solubility of methadone free base and methadone hydrochloride in solvents at 32 ± 1 °C and solubility parameters of solvents.

The solubility values for methadone hydrochloride in water and ethanol have been reported previously as 120 and 80 mg/mL, respectively [28]. The solubility of methadone hydrochloride and free base at 32 ± 1 °C was measured in a range of solvents (Table 2). As expected, methadone hydrochloride is poorly soluble in solvents such as LIM, EO, IPM and OSAL and relatively high solubility was observed for hydrophilic solvents. The highest solubility of methadone hydrochloride was observed in PG. The solubility values for methadone free base and its hydrochloride salt in PBS are 0.49 ± 0.01 and 86.66 ± 0.95 mg/mL, respectively. The addition of 6% *w/v* Brij™ O20 in PBS increased the solubility of methadone free base and methadone hydrochloride to 2.61 ± 0.02 and 107.45 ± 1.35 mg/mL, respectively. Methadone free base has a calculated van Krevelen and Hoftyzer solubility parameter of 10.07 (cal/cm^3^)^1/2^ and its solubility in solvents with solubility parameters, ranging from 8 to 11 (cal/cm^3^)^1/2^ was >100 mg/mL (Figure 4). These results are consistent with the theory proposed by Hancock, et al. [29] i.e., when the solubility parameters of solute and solvent are similar, high solubility is expected compared with solvents that do not have similar solubility parameters. On the other hand, solubility parameters only provide an indication of solubility characteristics and cannot account for all circumstances. Molecules may take up different conformations in the various solvents and anomalies are possible. It is interesting to note that methadone base has relatively low solubility in hydrophilic solvents with solubility parameter value above 11 (cal/cm^3^)^1/2^ and it showed a lower solubility in TriPG compared with OSAL. The anomalous solubility behavior of caffeine was also reported previously [30,31,32].

### 3.5. In Vitro Permeation and Mass Balance Studies of Methadone Base in Porcine Ear Skin

**Figure 5:**
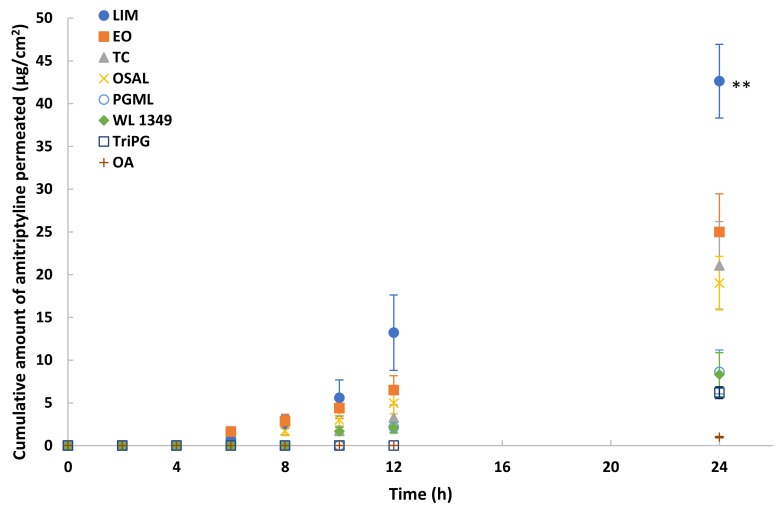
Cumulative permeation profiles of methadone in porcine skin after application of 5% (*w/v*) methadone free base in LIM (●), EO (■), TC (▲), OSAL (×), PGML (○), WL 1349 (♦), TriPG (☐) and OA (+) for finite dose (5 μL/cm^2^) at 32 ± 1 °C. Each data point represents the mean ± SD (mean ± SD; 4 ≤ *n* ≤ 5). ** *p* < 0.01.

Eight solvents were selected for in vitro permeation studies of methadone free base in porcine skin under finite dose conditions (5 μL/cm^2^). The permeation profiles are shown in Figure 5. The highest permeation of methadone was observed for LIM (*p* < 0.01). The cumulative amount of methadone that permeated after 24 h was 41.3 ± 4.7 μg/cm^2^ (20.7 ± 2.3% of the applied dose). The results also suggest EO, TC and OSAL are promising vehicles for methadone. Although methadone hydrochloride permeation in PG was investigated also, the maximum cumulative amount that permeated through porcine skin after 24 h was only 2.5 ± 0.6 μg/cm^2^.

**Figure 6:**
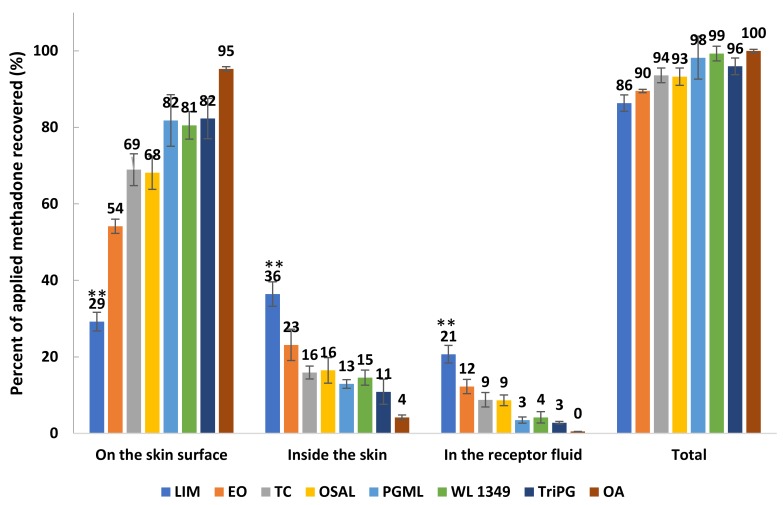
Mass balance results after 24 h finite dose (5 μL/cm^2^) permeation studies in porcine skin for 5% (*w/v*) methadone free base in LIM, EO, TC, OSAL, PGML, WL 1349, TriPG and OA at 32 ± 1 °C (mean ± SD; 4 ≤ *n* ≤ 5). ** *p* < 0.01.

Mass balance studies confirm that high permeation of methadone generally was associated with high amounts of drug extracted from the skin (Figure 6). Except for LIM, the overall recoveries for other formulations were within the recovery range (90–110%) recommended in the OECD guidelines [33]. Although the stability of methadone free base in formulations and the receptor fluid was confirmed, the overall recovery of methadone for LIM was only 86.33 ± 2.15%. However, LIM still delivered significantly higher (*p* < 0.05) percentages of methadone into the skin and through the skin compared with the other solvents. The results confirm that the lowest percentage of methadone recovered from the skin surface was observed for LIM, followed by EO, OSAL and TC (Figure 6). Except for LIM, the percentages of applied methadone recovered from the skin surface were significantly higher compared with the percentages of methadone inside skin and permeated across skin.

A proposed mechanism of penetration enhancement for LIM is the disruption of the highly ordered structure of intercellular lipids which may result in an increase in drug diffusion [34]. The permeation of LIM through human skin and high affinity of LIM for the epidermis were reported by Cal, et al. [35]. This indicates another potential mechanism that may contribute to high permeation of methadone in the present study. With the exception of OA, the highest solubility of methadone base was observed for LIM. The uptake of LIM into skin may create a more favorable environment for methadone and thus increase the partition of methadone into the stratum corneum (SC). In addition, both skin uptake and evaporation of LIM can also contribute to an increase in the thermodynamic activity of methadone and the partition into skin. On the other hand, drug crystallization inside the skin or on the skin surface may occur as well when LIM permeates through skin. LIM is generally recognized as a safe ingredient in topical products [36]; the highest concentration used in FDA approved topical products is 10% *w/w* [37]. 

EO is derived from the esterification of OA with ethanol. Both EO and OA have a double bond in the *cis* configuration, forming a “kinked” shape [38]. This kink conformation may disrupt SC lipid packing by introducing a separate phase in the intercellular lipid domain and thus increase the diffusion of solute through skin [39,40]. The permeation of methadone for OA was significantly lower than that for EO and other solvents (*p* < 0.05). This may reflect electrostatic interaction between methadone free base and the carboxylic acid group of OA. The presence of ionized methadone might also be expected to reduce the partition of methadone from OA into SC lipids, thus reducing the permeation of methadone. FTIR was subsequently used to confirm an interaction between methadone free base and OA (Figure S2). The high solubility of methadone in OA reported above also supports the possibility of an interaction between OA and methadone free base. 

The cumulative amount of methadone that permeated through skin for OSAL was significantly higher than for PGML and medium-chain triglycerides of caprylic and capric acids (WL 1349) (*p* < 0.05) while there is no significant difference between the amounts of methadone extracted from skin for these solvents (*p* > 0.05). This indicates that OSAL is more likely to enhance permeation by increasing the diffusion of methadone through skin rather than altering the partition of methadone into stratum corneum. Santos, et al. [41] reported that OSAL is taken up to a high extent by the skin but no effect on lipid fluidity was observed using DSC and FTIR. The authors proposed that OSAL may enhance drug diffusion by creating “pools” of solvent within the gel lipid phase.

The cumulative amount of methadone that permeated through porcine skin from TC after 24 h was 21.07 ± 5.12 μg/cm^2^. Almost double the amount of methadone was recovered following skin extraction compared with the amount that permeated for this vehicle. TC is miscible with both polar and non-polar solvents and it has been widely used as a penetration enhancer. Haque, et al. [42] reported that 53.4% of TC permeated through human skin in Franz cell studies by 19 h. The rapid skin uptake of TC suggest that TC is likely to enhance permeation by increasing drug solubility in intracellular lipids. TC may therefore play a key role in driving the partition of methadone into skin and increasing permeation. Increasing thermodynamic activity of methadone resulting from TC evaporation and skin uptake may also contribute to the skin permeation of methadone. The highest concentrations of TC, OSAL and EO used in FDA approved topical products are 49.91% *w/w*, 8.5% *w/w* and 0.14 mg/mL, respectively [37]. 

Fullerton et al. [13] evaluated the transdermal delivery of methadone free base using human cadaver skin over 24 h. Solutions of methadone base were prepared in ethanol and propylene glycol and in ethanol and propylene glycol with either oleic acid or Azone^®^. The highest amount of methadone permeated from the ethanol: PG: Azone solution (~400 μg/cm^2^), however the concentration of methadone (720 μg/μL) was more than 10-fold greater than that evaluated in the present work. Ghosh and Bagherian [14] also investigated a transdermal patch containing methadone free base in human skin. Patches with or without 5% *w/w* Azone^®^ or 5% *w/w n*-decylmethyl sulfoxide were tested. The cumulative amounts of methadone permeation at 24 h in human skin ranged from 1 to 3 mg/cm^2^. Again, comparisons with the present work are limited because of the comparatively larger doses of methadone in the patches compared with the formulations examined here. It is also important to note that the objectives of these two earlier studies were systemic delivery of methadone for management of substance abuse rather than local delivery. 

## 4. Conclusions

Methadone free base was prepared from its hydrochloride salt, followed by a series of characterization studies. A range of solvents was evaluated using in vitro permeation and mass balance studies in porcine skin and a maximum skin flux of 2.54 ± 0.19 μg/cm^2^/h was estimated. The results suggest that TC, LIM, OSAL and EO appear to be promising candidate vehicles for dermal delivery of methadone base. This is the first study to confirm skin permeation of methadone base under finite dose conditions. However, for most formulations the majority of the active remained on the skin surface after 24 h. Future work will focus on development of more complex vehicles with further optimization and evaluation of skin permeation in human skin. The most promising formulations will be progressed for investigation in the clinic. 

## Figures and Tables

**Table 1:** Distribution coefficient of methadone for different pH values at 25 ± 1 °C.

pH	Log D
6.0	0.31 ± 0.01
6.5	0.80 ± 0.01
7.0	1.35 ± 0.01
7.4	1.82 ± 0.01
11.0 (free base)	4.85 ± 0.03

**Table 2:** Summary of the solubility of methadone hydrochloride and free base in neat solvents at 32 ± 1 °C. The solubility parameter of methadone free base is 10.07 (cal/cm^3^)^1/2^.

Solvent	Solubility of Methadone base (mg/mL)	Solubility of Methadone Hydrochloride (mg/mL)
LIM	342.38 ± 2.99	0.08 ± 0.002
EO	205.46 ± 1.69	0.08 ± 0.01
IPM	179.80 ± 0.89	0.03 ± 0.001
WL 1349	217.66 ± 3.73	0.05 ± 0.001
OA	438.76 ± 2.56	2.53 ± 0.03
PGML	209.43 ± 0.54	19.68 ± 0.79
PGMC	250.74 ± 2.17	28.65 ± 0.46
TC	247.68 ± 0.95	21.96 ± 0.46
OSAL	277.20 ± 6.96	0.05 ± 0.001
TriPG	105.90 ± 0.25	18.03 ± 0.93
DiPG	85.94 ± 0.41	43.97 ± 1.28
PG	24.78± 0.49	201.33 ± 1.30
1,3-BD	26.24 ± 0.05	88.40 ± 0.79
PBS	0.49 ± 0.01	86.66 ± 0.95
PBS + 6% *w/v* Brij™ O20	2.61 ± 0.02	107.45 ± 1.35

## Supplementary Materials

The following are available online at https://www.mdpi.com/1999-4923/11/10/509/s1, Figure S1: IR spectra of (A) methadone free base and (B) methadone hydrochloride, Figure S2: IR spectra of (A) oleic acid and (B) 10mg methadone free base dissolved in 0.1 mL oleic acid (OA), Figure S3: Thermogravimetric analysis of methadone hydrochloride and methadone free base, Figure S4: Recovery (%) of methadone free base in a series of neat after 24, 48, 72 and 96 h at 32 ± 1 °C (*n* = 3; mean ± SD).

## Abbreviations

1,3-BD	1,3-butanediol
^1^H-NMR	Proton Nuclear Magnetic Resonance
ANOVA	One-way Analysis of Variance
CDCl3	Chloroform-d
DiPG	Dipropylene Glycol
DSC	Differential Scanning Calorimetry
EO	Ethyl Oleate
FTIR	Fourier Transform Infrared
GRAS	Generally Recognized as Safe
HPLC	High Performance Liquid Chromatography
IPM	Isopropyl Myristate
LIM	d-limonene
LOD	Limit of Detection
Log D	Logarithm to the Base 10 of Distribution Coefficient
Log P	Logarithm to the Base 10 of Partition Coefficient
LOQ	Limit of Quantification
Nav	Voltage-gated Sodium (Nav) Channels
NMDA	*N*-Methyl-d-aspartate
OA	Oleic Acid
OECD	Organisation for Economic Co-operation and Development
OSAL	Octyl Salicylate
PBS	Phosphate Buffered Saline
PG	Propylene Glycol
PGMC	Propylene Glycol Monocaprylate
PGML	Propylene Glycol Monolaurate
pK_a_	Dissociation Constant
SD	Standard Deviation
TC	Transcutol^®^
TGA	Thermogravimetric Analysis
TriPG	Tripropylene Glycol
UV	Ultraviolet
WL 1349	Labrafac™ lipophile WL 1349
