# Supplementary material for: Preparation, Characterization and Dermal Delivery of Methadone
Source: Pharmaceutics. 2019 Oct 2;11(10):509. doi: 10.3390/pharmaceutics11100509 (PMC6835921; doi:10.3390/pharmaceutics11100509)
Supplement: Supplementary file 1 [file pharmaceutics-11-00509-s001.pdf]

pharmaceutics
MDPI

Supplementary Materials: Preparation, Characterization and Dermal Delivery of Methadone

Chin-Ping Kung, Bruno C. Sil, Jonathan Hadgraft, Majella E. Lane, Bhumik Patel and Renée McCulloch

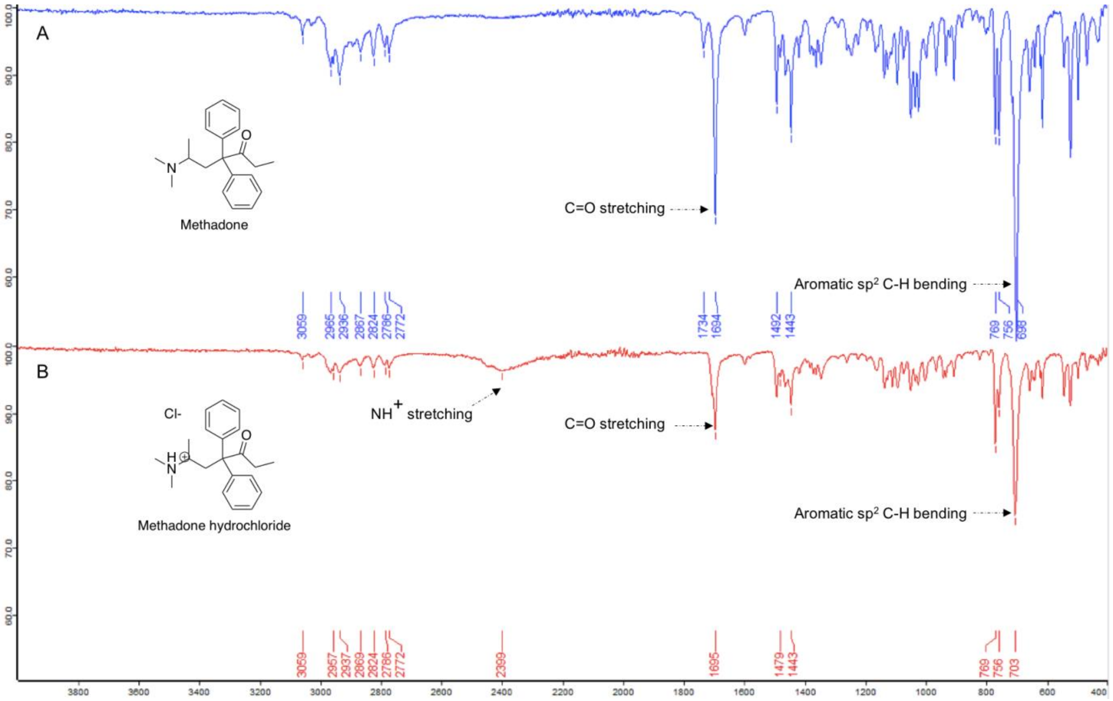

Figure S1. IR spectra of (**A**) methadone free base and (**B**) methadone hydrochloride. Data was reported as percent transmission (%T). The spectra were obtained with a Bruker Alpha Spectrometer with a Platinum ATR accessory (Bruker, UK). High absorption signals at 698 cm^{-1} and 1694 cm^{-1} were assigned to the aromatic sp^2 C-H bending and ketone C=O stretching for methadone. A broad band at 2399 cm^{-1} which was assigned to NH+ stretching was observed for methadone hydrochloride while there was no such band found for methadone hydrochloride [1].

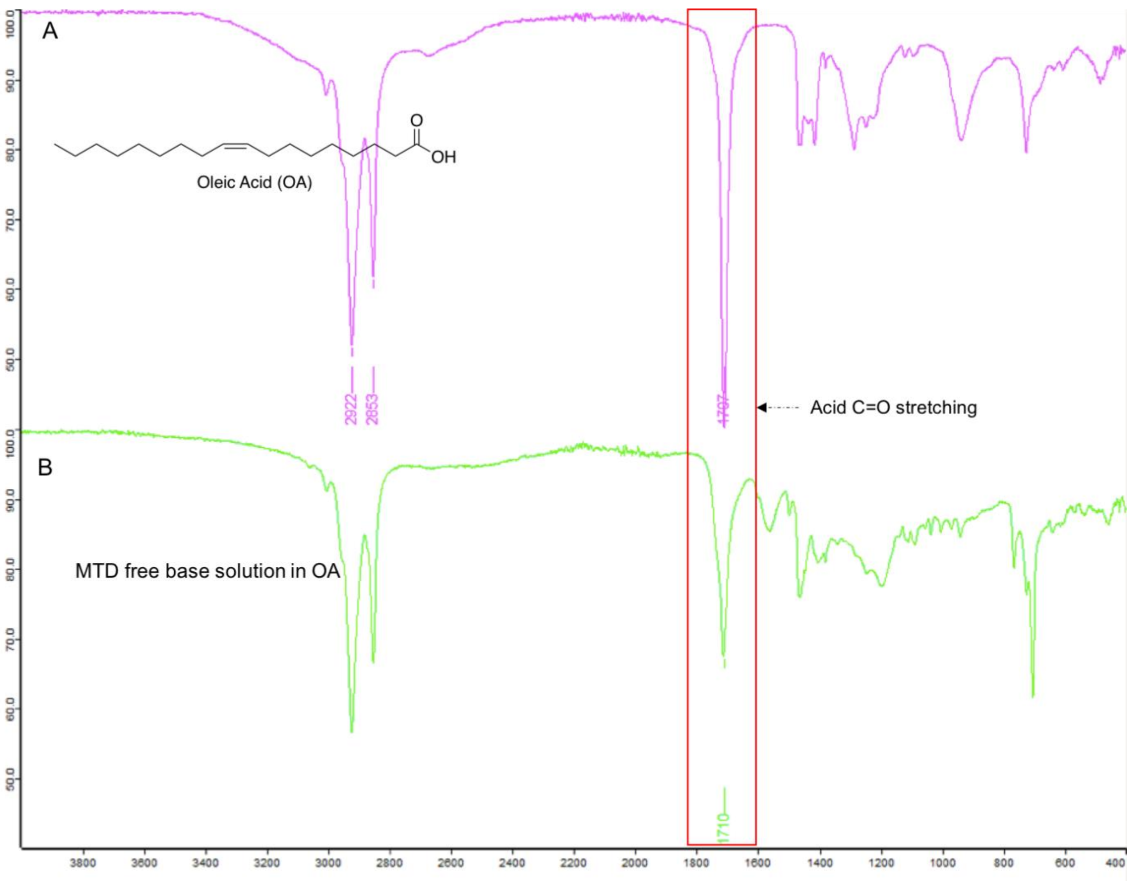

Figure 2. IR spectra of (**A**) oleic acid and (**B**) 10mg methadone free base dissolved in 0.1 mL oleic acid (OA). Data was reported as percent transmission (%T). The spectra were obtained with a Bruker Alpha Spectrometer with a Platinum ATR accessory (Bruker Optics, Coventry, UK). A peak at 1707 cm^{-1} was assigned to the carboxylic acid C=O stretching of oleic acid [1]. The absorbance of this peak for methadone solution in oleic acid is lower than that for neat oleic acid. This confirms the electrostatic interaction between methadone free base and the carboxylic acid group of oleic acid.

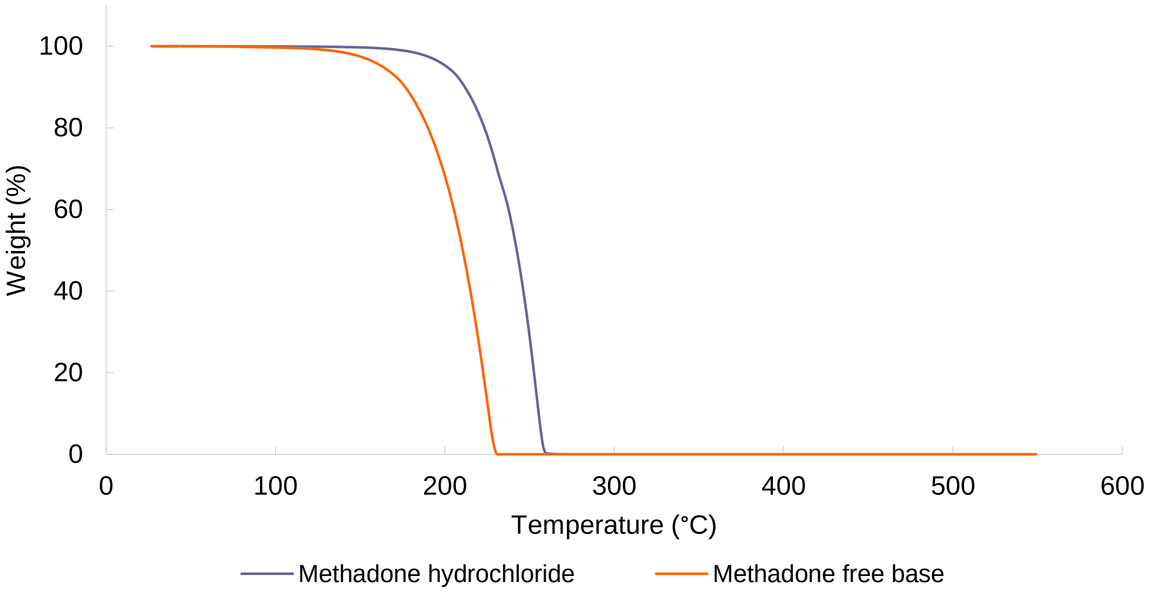

Figure 3. Thermogravimetric analysis of methadone hydrochloride and methadone free base.

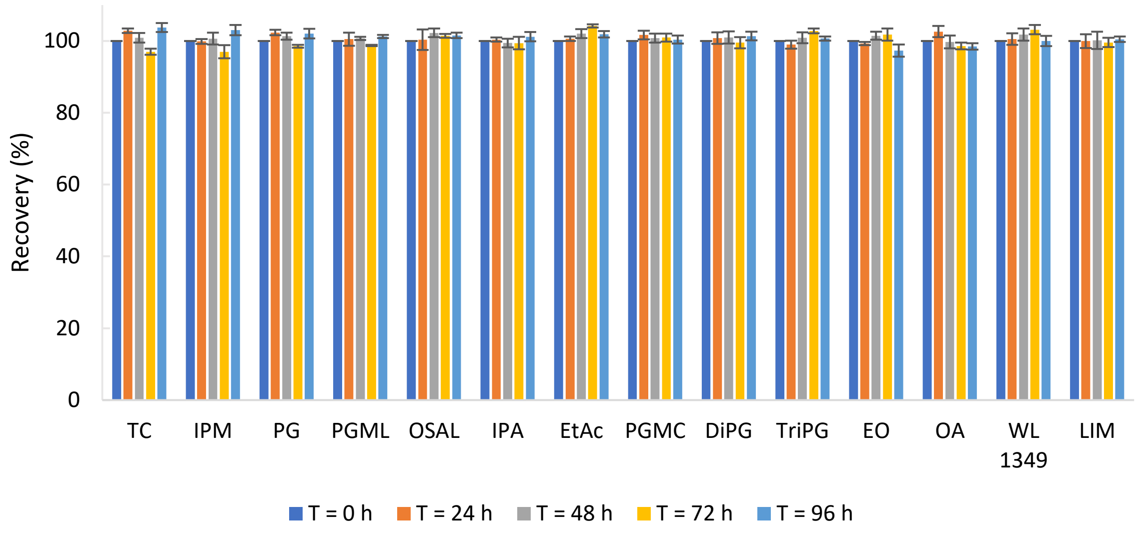

Figure 4. Recovery (%) of methadone free base in a series of neat after 24, 48, 72 and 96 h at 32 ± 1 °C (n = 3; mean ± SD).

References:

1. Coates, J. Interpretation of infrared spectra, a practical approach. In *Encyclopedia of analytical chemistry: applications, theory and instrumentation*, 2000.
